# Water Vapor Pressure Deficit in Portugal and Implications for the Development of the Invasive African Citrus Psyllid *Trioza erytreae*

**DOI:** 10.3390/insects11040229

**Published:** 2020-04-07

**Authors:** Paulo Eduardo Branco Paiva, Tânia Cota, Luís Neto, Celestino Soares, José Carlos Tomás, Amílcar Duarte

**Affiliations:** 1 Instituto Federal de Educação, Ciência e Tecnologia do Triangulo Mineiro (IFTM), 38064-790 Uberaba, Brazil; paulopaiva@iftm.edu.br; 2 Divisão de Clima e Alterações Climáticas, Departamento de Meteorologia e Geofísica, Instituto Português do Mar e da Atmosfera I. P. (IPMA, IP), Rua C do Aeroporto, 1749-077 Lisboa, Portugal; tania.cota@ipma.pt; 3 MED - Mediterranean Institute for Agriculture, Environment and Development, University of Algarve, 8005-139 Faro, Portugal; lneto@ualg.pt; 4 Direção Regional de Agricultura e Pescas do Algarve, Patacão, 8001-904 Faro, Portugal; cbsoares@drapalgarve.gov.pt (C.S.); jctomas@drapalgarve.gov.pt (J.C.T.)

**Keywords:** greening, Huanglongbing, *Candidatus* Liberibacter africanus, Triozidae, abiotic factor limitations

## Abstract

African citrus psyllid (*Trioza erytreae* (Del Guercio)) is a vector insect of the bacterium *Candidatus* Liberibacter africanus, the putative causal agent of Huanglongbing, the most devastating citrus disease in the world. The insect was found on the island of Madeira in 1994 and in mainland Portugal in 2015. Present in the north and center of the country, it is a threat to Algarve, the main citrus-producing region. *Trioza erytreae* eggs and first instar nymphs are sensitive to the combination of high temperatures and low relative humidity. Daily maximum air temperature and minimum relative humidity data from 18 weather stations were used to calculate the water vapor pressure deficit (vpd) from 2004 to 2018 at various locations. Based on the mean vpd and the number of unfavorable days (vpd < 34.5 and vpd < 56 mbar) of two time periods (February to May and June to September), less favorable zones for *T. erytreae* were identified. The zones with thermal and water conditions like those observed in the Castelo Branco and Portalegre (Center), Beja (Alentejo), Alte, and Norinha (Algarve) stations showed climatic restrictions to the development of eggs and first instar nymphs of African citrus psyllid. Effective control measures, such as the introduction and mass release of *Tamarixia dryi* (Waterson), a specific parasitoid, and chemical control are necessary in favorable periods for *T. erytreae* development, such as in spring and in areas with limited or no climate restrictions.

## 1. Introduction

African citrus psyllid (*Trioza erytreae* (Del Guercio)) is a vector insect of the bacterium *Candidatus* Liberibacter africanus, the putative causal agent of Huanglongbing (HLB), also known as Greening, which is the most devastating citrus disease in the world. This species was discovered on the island of Madeira in 1994 and on the Canary Islands in 2002, and was found in continental Europe in Spain in 2014 and mainland Portugal in 2015 [1,2,3]. So far, the bacteria associated with HLB have not been found in Europe, but the presence of the insect vector makes an eventual accidental introduction of the bacteria a looming disaster for European citrus production. To prevent the arrival of the insect at the main citrus-producing areas of these countries, efforts to contain the insect by establishing containment plans including restrictions on the transit of plant material have been conducted [3]. Despite all the preventive measures included in these containment plans, *T. erytreae* spread widely in the coastal region of Portugal [4]. 

The African HLB pathosystem (HLBaf) associated with the bacterium *Candidatus* Liberibacter africanus (CLaf) transmitted by *T. erytreae* is climate-dependent and is intolerant to high temperatures [5,6]. In South Africa, the insect and CLaf find the best conditions at high altitudes in cold and humid areas [6,7,8,9]. Catling [10] showed that hot and dry days with high water vapor pressure deficits (vpd) were lethal to *T. erytreae* eggs and first instar nymphs. These adverse conditions determined the seasonal abundance and geographical distribution of the insect [11,12,13]. There was a significant inverse correlation between the occurrence of *T. erytreae* and days with vpd greater than 25.9 mmHg (34.5 mbar), a condition causing 70% mortality of *T. erytreae* eggs and nymphs [11,12,13,14]. Green and Catling [8] related vpd to survival of *T. erytreae* early life stages and found that no survival was observed when the vpd was above 56 mbar.

Thus, areas with prolonged periods of high vpd may be unsuitable for the development of the early stages of *T. erytreae*, thereby limiting population growth. The southern region of Portugal, the country’s main citrus-producing zone, has a temperate climate with a dry, hot summer (Csa) [14] and low altitudes. Using historical climatic data from 2004 to 2018 from 12 national weather stations distributed throughout mainland Portugal and from six regional weather stations from southern Portugal (Algarve), we determined which periods in what regions the weather conditions would be unfavorable or even lethal for the early stages of *T. erytreae*. Water vapor pressure deficit (vpd) was assessed alongside two important abiotic factors, temperature and air humidity. Using vpd values obtained from the network of stations included in this study, less favorable areas and periods for the development of *T. erytreae* were able to be predicted. This information is important in the building of forecasting models, the establishment of risk zones, and the adaptation of monitoring procedures and pest control tactics to distinct zones.

## 2. Materials and Methods

**Figure 1:**
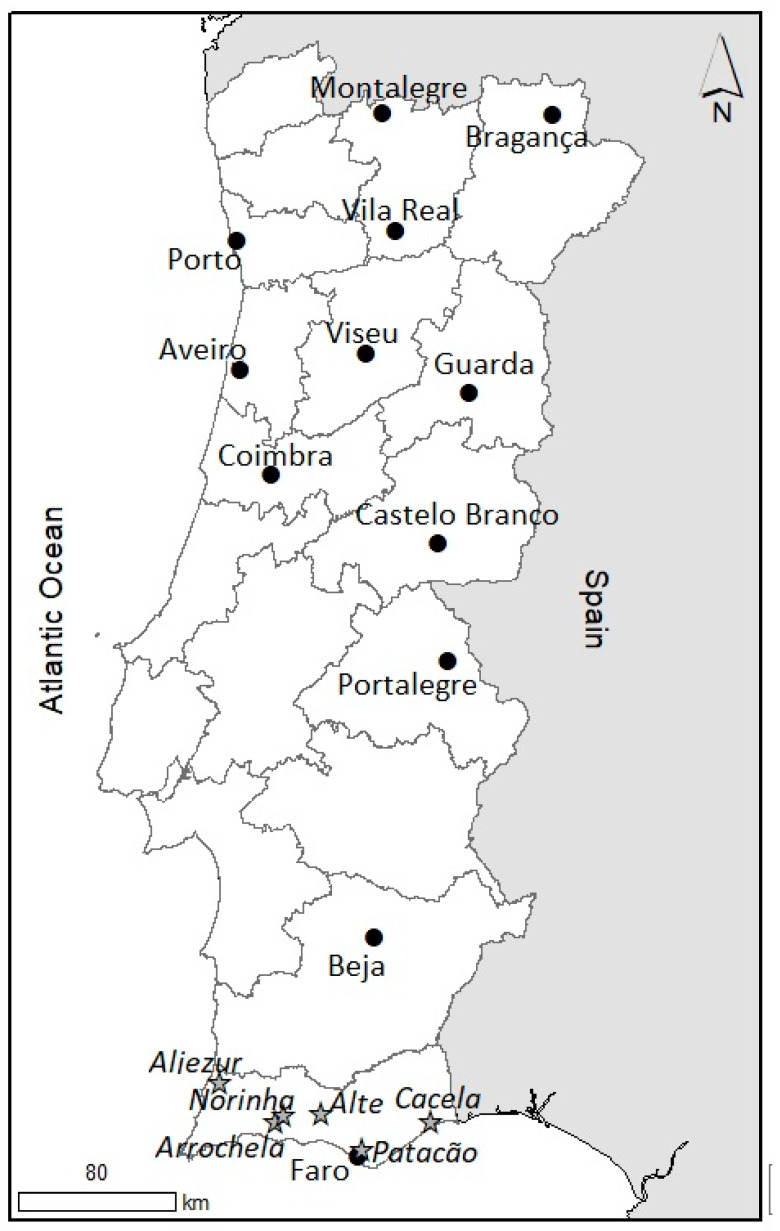
Portuguese Institute of the Sea and Atmosphere (black circles, IPMA) and Regional Directorate for Agriculture and Fisheries of Algarve (stars, DRAP Algarve) weather stations.

Daily data on maximum temperature (t_max_ in °C) and minimum relative humidity (RH_min_ in %) were obtained from 12 automatic Portuguese Institute of Sea and Atmosphere (IPMA) weather stations and six Regional Directorate for Agriculture and Fisheries of Algarve (DRAP Algarve) weather stations from 2004 to 2018 (Table 1, Figure 1). Using these climate variables, the daily vapor pressure deficit (vpd) was calculated according to the formula vpd=sp−ap, where saturation pressure was $sp=\left(6.1078\times 10^{7.5\times tmax}\right)÷\;\left(237.5+tmax\right)$ [15] and the current pressure was ap=sp×RHmin .

The monthly vpd was calculated for each season from February to September of each year using daily vpd data. The data were organized into two four-month periods. The first period, from February to May, covered the beginning of citrus flush and the months of the spring season, including bloom and the development of the leaves that accompany fruiting. The second period, from June to October, included the summer months and September, covering the later flush. These two periods covered the full span of when the trees had the young leaves necessary for egg laying and the development of immature *T. erytreae*.

For both periods, the number of days with vpd higher than 34.5 mbar (= 25.9 mm Hg) and the number of days with vpd higher than 56 were calculated, with the conditions leading to greater than 70% mortality of *T. erytreae* eggs and first instar nymphs, respectively, [10] and the total mortality of these developmental stages [10]. Green and Catling [8] proposed the following equation to estimate *T. erytreae* survival (s) according to the variable vpd, i.e., $s=137.7709-\left(4.1825\times vpd\right)+\left(0.0308\times vpd^{2}\right)\;$.

Daily data regarding the maximum temperature and minimum relative humidity of 12 national IPMA stations and six DRAP Algarve stations were statistically analyzed considering the season as the treatment and the year as the replicate (n = 15). Data that met the parametric analysis assumptions were subjected to analysis of variance; the means were clustered using the Scott–Knott method (*p* < 0.05). The other data were submitted to deviance analysis using generalized linear models (Poisson distribution) and generalized Tukey’s test (*p* < 0.05). Analyses were performed using R (version 3.5.3) software [16]. Box-plot graphs were generated for the vpd data. The numbers of days with vpds higher than 34.5 or 56 mbar were tabulated.

Daily vapor pressure deficit data and the number of days spent above 34.5 and above 56 mbar were determined for all stations. IPMA stations were organized according to the agrarian regions, i.e., North (Bragança, Montalegre, Porto and Vila Real), Center (Aveiro, Castelo Branco, Coimbra, Guarda and Viseu), Lisbon and Tagus Valley (Portalegre), Alentejo (Beja), and Algarve (Faro). DRAP Algarve stations followed the regional division of the Algarve: Atlantic Coast (Aljezur), Barlavento (Arrochela, Norinha, and Alte), and Sotavento (Patacão and Cacela).

## 3. Results

**Figure 2:**
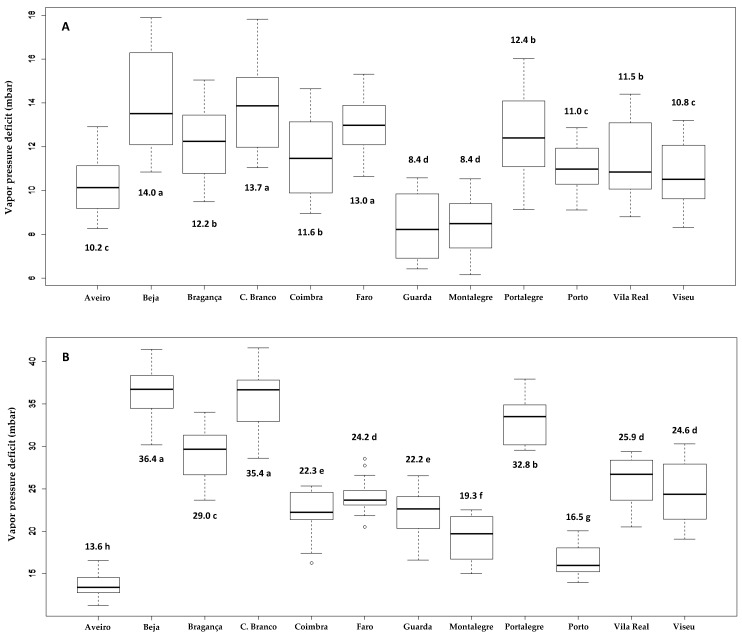
Box-plot graphs of mean water vapor pressure deficits in twelve areas of mainland Portugal in the spring (**A**) (February to May) and summer (**B**) (June to September) from 2004 to 2018 (ANOVA: February to May, F = 17.04, *p* < 0.001; June to September, F = 91.00, *p* < 0.001). Highlighted means with the same letters were not different by Scott–Knott grouping test (*p* < 0.05).

From February to May, the lowest mean vpds were observed in Aveiro, Guarda, and Montalegre, and the highest vpds were observed in Beja and Portalegre. The other zones showed intermediate vpds. From June to September, Aveiro and Porto exhibited the lowest vpds, with the highest vpds found in Beja, Castelo Branco, and Portalegre (Figure 2).

**Figure 3:**
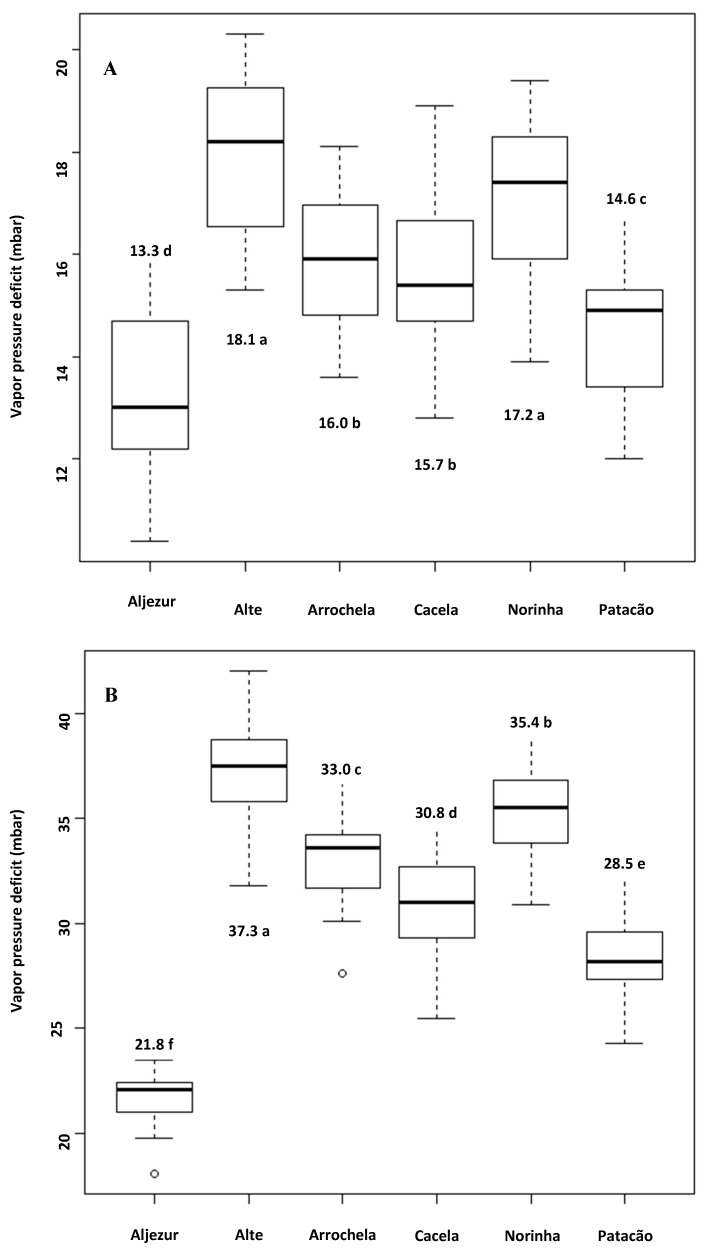
Box-plot graphs of mean water vapor pressure deficits in six zones of Algarve in the spring (**A**) (February to May) and summer (**B**) (June to September) from 2004 to 2018 (ANOVA: February to May, F = 17.91, *p* < 0.001; June to September. F = 84.00, *p* < 0.001). Highlighted means with the same letters were not different by Scott–Knott grouping test (*p* < 0.05).

In the Algarve, the lowest mean vpd was found in Aljezur in both periods analyzed. The highest vpds were observed in Alte and Norinha, the inland stations. No differences were observed between the stations from February to May, but the highest vpd occurred in Alte from June to September (Figure 3).

There were few days with vpd above 34.5 mbar in the period from February to May at the IPMA stations. The highest mean was obtained at Beja station, with 4.2 days after 4 months. In the four-month period from June to September, more days were observed in this condition in the Castelo Branco (65.7 days), Beja (58.6 days), and Portalegre regions (54.4 days), amounting to almost half of the analyzed period (Table 2). No observations were made of vpd above 56 mbar from February to May at the national stations. From June to September, Beja showed 9.5 days, Castelo Branco showed 6.1 days, and Portalegre showed 4.5 days at these extreme conditions.

In the Algarve, Alte and Norinha spent the most days with vpd greater than 34.5 mbar during the February–May period. From June to September, the Alte station showed 69.4 days with vpd above 34.5 mbar. The second station with the most days with vpd above 34.5 mbar was Norinha. Readings over 56 mbar were observed on 9.3 days in Alte, 6.2 days in Norinha, and 5.3 days in Arrochela (Table 2).

**Figure 4:**
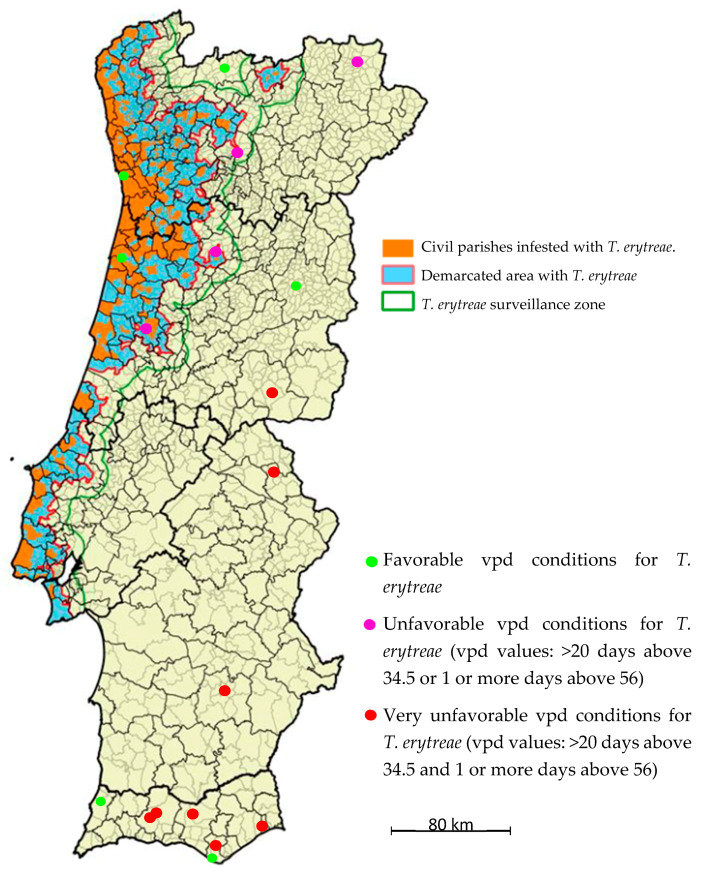
Distribution map of *Trioza erytreae* in mainland Portugal. The delimitation of the “Infested Zone” is based on the civil parishes where *T. erytreae* was detected. A “buffer zone” was added to this zone, surrounding a 3 km radius considering the flight capacity of the insect. A “Surveillance Zone” of 10 km radius was also defined around the Demarcated Zone (Infested Area + Buffer Zone). Available online by the General Directorate of Food and Veterinary of the Portuguese Ministry of Agriculture [4] (http://www.dgv.min-agricultura.pt/portal/page/portal/DGV/genericos?generico=221911&cboui=221911).

Stations were classified into three categories according to their vpd conditions for *T. erytreae* survival: 1) favorable conditions; 2) unfavorable vpd conditions (vpd values: >20 days above 34.5 or one or more days above 56); and 3) very unfavorable vpd conditions (vpd values: >20 days above 34.5 and one or more days above 56). These stations were marked on the map (Figure 4) with green and white and blue and red circles, respectively. As yet, the expansion of *T. erytreae* as monitored by the Directorate General for Food and Veterinary of the Portuguese Ministry of Agriculture [4], marked in orange in Figure 4, has not reached any of the zones classified as very unfavorable; only one case has come close to an unfavorable zone so far.

## 4. Discussion

The most adverse zones for insect development are Beja, Castelo Branco, Portalegre, Alte, and Norinha based on the variables we evaluated that indicated lethal days for early stages of *T. erytreae*. The five zones where the insect was present, i.e., Montalegre and Porto and Vila Real in the north and Aveiro and Coimbra in central Portugal [4], were favorable to *T. erytreae* due to low vpd and few lethal days. In the Algarve, Aljezur was the most suitable zone for the insect because there was practically no limitation of the development of eggs and early nymphs. Extremely adverse days to psyllid development during June to September with vpd above 56 mbar occurred in Beja (9.5 days) and Alte (9.3 days).

Very hot and dry days, represented by the highest vpd levels (i.e., >34.5 mbar) may not control the African HLB vector, but they could be an abiotic factor that limits population outbreaks from June to September. The number of lethal days found in Beja Alte and Norinha were similar to those in Letaba (South Africa) in 1965/67, where *T. erytreae* populations were rarely found in the summer months [10]. Further, the occurrence of lethal days and irregular flushing in South Africa were responsible for low *T. erytreae* densities in 1968–1970 [17]. However, adults are not susceptible to high vpd [11]. The succession of eight lethal days caused the mortality of susceptible life stages (eggs and first instar nymphs) in Nelspruit in 2012, but not in adults [18]. Thus, the absence of adverse spring days may allow adults to develop and transmit the disease during this season, as well as in summer. The high longevity of adults from 17 to 50 days [19] with availability of summer flush allows *T. erytreae* to stay in citrus [20].

In southern Portugal, citrus flush begins in February with bloom, allowing plenty of feeding and oviposition sites for *T. erytreae*. During this period there are no lethal days and the insect population may increase. In summer, despite the availability of flush, the survival of eggs and early instars may be difficult. The density of *T. erytreae* is higher in the highlands [20] but Portugal’s commercial citrus industry is in the lowlands with hot and dry summers, which can be aggravated by increasing climatic extremes [21]. The insect might be more of a pest in climatically favorable areas if host plants are available.

Citrus trees are widely distributed in noncommercial and urban areas in Portugal. Sour orange (*Citrus aurantium* L.) and lemon trees (*Citrus limon* (L.) Burm. f.) in backyards and along roadways are very common in Portugal [22]. Elimination of Rutaceae host plants, such as *Clausena anisata* (Willd.), near citrus has been recommended [23]. *Murraya koenigii* (L.) was the best breeding host of *T. erytreae* among those tested by Aidoo et al. [24], thus. it may also be a candidate for regulatory action.

In the colder months of the year from November to February, it is plausible that the insect will find the low temperature conditions too adverse. The minimum base temperature for *T. erytreae* is 10–12 °C [19]. Therefore, control measures, such as classical biological control with the introduction of *Tamarixia dryi*, an efficient and specialist parasitoid [24,25,26], and chemical control (mainly with neonicotinoid insecticides), may be focused on spring flush when the weather is favorable to insect survival and development. With warmer summers, smaller populations of *T. erytreae* are expected; however the insect should be sampled regularly for the presence of CLaf and controlled in an area-wide manner [27]. Warm and dry areas are less favorable to *T. erytreae* [28] and may be more suitable for citrus production in Portugal in the long run. If HLB is introduced in Portugal, citrus-production should move to inland areas of the Algarve, which are less favorable to the HLBaf pathosystem (CLAf and *T. erytreae*), thereby potentially facilitating the management of the disease. 

## 5. Conclusions

Based on the water vapor pressure deficit (vpd) monthly means and number of days above 34.5 and 56 mbar, the period from February to May (spring) is more suitable to *T. erytreae* than the period from June to September (summer) in Portugal. In the zones of Castelo Branco, Portalegre (Center), Beja (Alentejo), Alte, and Norinha (Algarve), the early developmental stages of *T. erytreae* may be affected negatively by climatic limitations and their development on warmer and drier days may be limited.

## Figures and Tables

**Table 1:** Location of the Portuguese Institute of the Sea and Atmosphere (IPMA) and Regional Directorate for Agriculture and Fisheries of Algarve (DRAP Algarve) weather stations and presence of *Trioza erytreae* in the region.

Zone	Location	Presence of	Inland	Geographical Coordinates		Altitude
		*T. erytreae*	or Coast	North-N	West-W	(masl ^1^)
**IPMA Stations**						
North	Bragança		inland	41°48′13,9″	06°44′34,2″	690
	Montalegre	X	inland	41°49′22,0″	07°47′16,4″	1005
	Porto	X	coast	41°13′56,2″	08°40′44,8″	69
	Vila Real	X	inland	41°16′27,1″	07°43′01,6″	561
Center	Aveiro	X	coast	40°38′07,4″	08°39′34,6″	5
	Castelo Branco		inland	39°50′21,9″	07°28′43,3″	386
	Coimbra	X	inland	40°09′27,4″	08°28′06,7″	171
	Guarda		inland	40°31′42,8″	07°16′43,2″	1020
	Viseu		inland	40°42′53,7″	07°53′45,3″	636
Lisbon and Tagus Valley	Portalegre		inland	39°17′39,1″	07°25′16,7″	597
Alentejo	Beja		inland	38°01′32,6″	07°52′02,3″	246
Algarve	Faro		coast	37°00′59,7″	07°58′19,0″	8
**DRAP Algarve stations**						
Atlantic coast	Aljezur		inland	37°21′24,9″	08°46′19,5″	91
Barlavento	Arrochela		inland	37°10′32,9″	08°26′48,1″	50
	Norinha		inland	37°12′19,7″	08°24′23,5″	15
	Alte		inland	37°12′40,8″	08°10′54,6″	79
Sotavento	Patacão		coast	37°02′48,8″	07°56′49,8″	13
	Cacela		coast	37°10′08,6″	07°33′08,2’	37

^1^ Meters above sea level.

**Table 2:** Mean number of days with water vapor pressure deficits (vpd) above 34.5 and 56.0 mbar in areas of mainland Portugal in the spring and summer from 2004 to 2018.

Zone	Location	Spring (February to May)				Summer (June to September)			
		Vpd > 34.5		Vpd > 56		Vpd > 34.5		Vpd > 56	
North	Bragança	0.6	c	0.00		37.2	b	0.7	c
	Montalegre	0.1	c	0.00		8.1	e	0.0	c
	Porto	0.7	c	0.00		8.2	e	0.1	c
	Vila Real	0.9	c	0.00		28.7	c	0.5	c
Center	Aveiro	0.7	c	0.00		4.9	f	0.2	c
	Castelo Branco	2.3	b	0.00		65.7	a	6.1	b
	Coimbra	1.6	c	0.00		15.9	d	1.1	c
	Guarda	0.0	c	0.00		15.3	d	0.0	c
	Viseu	0.4	c	0.00		26.7	c	0.6	c
Lisbon and Tagus Valley	Portalegre	2.3	b	0.00		54.4	a	4.5	b
Alentejo	Beja	4.2	a	0.10		58.6	a	9.5	a
Algarve	Faro	1.1	c	0.00		17.3	d	0.5	c
deviance		185.41 ^2^				2551.20 ^2^		632.22 ^2^	
p-valor		<0.001				<0.001		<0.001	
Algarve									
Atlantic coast	Aljezur	2.2	b	0.0	a	11.8	f	0.8	d
Barlavento	Arrochela	2.9	b	0.1	a	51.8	c	5.3	b
	Norinha	5.1	a	0.1	a	61.2	b	6.2	b
	Alte	7.3	a	0.1	a	69.4	a	9.3	a
Sotavento	Patacão	1.8	b	0.1	a	32.3	e	1.7	cd
	Cacela	2.9	b	0.1	a	43.8	d	3.1	c
F		7.05 ^1^				100.73 ^1^			
deviance				2.77 ^2^				178.36 ^2^	
p-value		<0.001		0.735		<0.001		<0.001	

^1^ Analysis of variance by the F test and means followed by the same letter in the column did not differ according to the Scott–Knott clustering test (*p* < 0.05). ^2^ Deviance analyses with generalized linear models and means followed by the same letter in the column did not differ by the generalized Tukey’s test (*p* < 0.05).
